# Nutritional properties of the largest bamboo fruit *Melocanna baccifera* and its ecological significance

**DOI:** 10.1038/srep26135

**Published:** 2016-05-19

**Authors:** Balaji Govindan, Anil John Johnson, Sadasivan Nair Ajikumaran Nair, Bhaskaran Gopakumar, Karuna Sri Lakshmi Mallampalli, Ramaswamy Venkataraman, Konnath Chacko Koshy, Sabulal Baby

**Affiliations:** 1 Phytochemistry and Phytopharmacology Division, Jawaharlal Nehru Tropical Botanic Garden and Research Institute, Pacha-Palode, Thiruvananthapuram 695 562 Kerala, India; 2 Plant Genetic Resources Division, Jawaharlal Nehru Tropical Botanic Garden and Research Institute, Pacha-Palode, Thiruvananthapuram 695 562 Kerala, India; 3 Centre for Lipid Research, CSIR-Indian Institute of Chemical Technology, Hyderabad 500 607, India; 4 Department of Chemistry, Sri Paramakalyani College (Manonmaniom Sundaranar University, Tirunelveli), Alwarkurichi 627 412, Tamil Nadu, India

## Abstract

*Melocanna baccifera* is a unique bamboo which produces the largest fruits in the grass family. Its gregarious flowering once in 45–50 years in north east India and adjacent regions is a botanical enigma, resulting in a glut of fruits. Proper utilization of *M. baccifera* fruits is not extant, and huge quantities of fruits are left underexploited due to lack of scientific information on their chemical composition and nutritional potential. Here we report the nutritional properties of *M. baccifera* fruits, and the ecological significance of its fruiting. This pear-shaped, fleshy bamboo fruit is rich in amino acids (lysine, glutamic acid), sugars (sucrose, glucose, fructose) and phenolics (ferulic acid). Protein content (free, bound) in *M. baccifera* fruits is very low. Fruits are rich in saturated fatty acids (palmitic acid), minerals (potassium), and only B series vitamins (B3) are detected in them. Rat feeding experiments showed that *M. baccifera* fruit alone is not a complete food, but with other protein supplements, it is a valuable food additive. This study could lead to better utilization of *M. baccifera* fruits during future flowering/fruiting events. These results could also help in the successful management of rodent outbreaks and other ecological problems associated with *M. baccifera* fruiting.

*Melocanna baccifera* (Roxb.) Kurz, belonging to the grass family Poaceae, is a unique bamboo naturally distributed in north east India, Nepal, Myanmar and Bangladesh and cultivated in several gardens world over^1^,^2^. *M. baccifera* is locally known as ‘Muli’ bamboo, and it has prime economical as well as ecological significance. It spreads aggressively and dominates over other vegetation in a short time^3^,^4^. Over 150 million people depend on this bamboo for making homes, agricultural supplements, mats, baskets, paper and pulp and its young shoots are used as food. Flowering of *M. baccifera* is reported to occur once in forty five to fifty years (monocarpic), and is in synchrony with populations which are distributed over a wide geographical area^2^. This gregarious flowering or mast flowering is a botanical enigma not so common in the plant kingdom, but is characteristic of woody bamboos^5^. *M. baccifera*, just like other woody bamboos, dies after flowering and fruiting. Its fruits are the largest among all bamboos (also in the grass family), pear-shaped, avocado like, green, fleshy, single seeded (caryopsis) and weighing up to 300 g^2^ (Fig. S1). The quantity of fruits produced from the culms of *M. baccifera* is enormous reaching up to 83.6 tons per hectare^6^ when compared to other bamboo fruits like *Bambusa bambos* and *Dendrocalamus strictus*^5^ which accounted only 36 tons/ha^5^,^7^. Recent flowering of *M. baccifera* from 2004-‘08 occurred in 1.76 m ha geographical area in north east Indian states (Mizoram, Tripura, Manipur, Meghalaya, Assam), and about 26 mt of Muli bamboo flowered and died^3^. The huge amount of fruits produced on *M. baccifera* flowering inevitably attracts various animals (mainly rodents), and ultimately leads to rodent outbreaks. Due to rapid life cycle and faster reproductive maturity, rodents (rats) colonize and start multiplying in quick succession when the food resource (fruits) is abundant. The fruiting season usually ranges between 2–3-(5) years and the increase in rodent population in this period is enormous^8^. When the fruiting season is over (or even during the fruiting season), the rodent population migrates to local villages, starts feeding on the rice and maize fields, results in heavy food loss and famine, and these events inflicted significant human mortality in the past^6^,^9^,^10^. Muli flowering and associated events are locally known as ‘*Mautam*’ (meaning ‘bamboo death’) and flowering-induced famine in 1959 claimed 10,000 to 15,000 lives throughout north east India^3^. The aggressive fruit feeding by rodents and the reason for their population outbreak following the mast flowering of this bamboo are not scientifically explained so far.

*M. baccifera* fruits are eaten as food by the local tribal people^5^ and also used as a medicine against low blood pressure^11^. There are only very few reports on the nutritional, chemical and medicinal aspects of *M. baccifera* fruits, possibly due to their infrequent (once in 45–50 years) occurrence. One such preliminary report indicated 50.3% starch, 11.6% protein, 0.2% fat and 3.0% ash in *M. baccifera* fruits^8^. A recent phytochemical study on *M. baccifera* fruits led to the isolation of olean-12-en-28-carboxy-3β-acetate, β-sitosterol glucoside, stigmasterol glucoside, 3-oxo-olean-12-en-28-al, isochavicinic acid and eugenol^12^. Another study reported significant DPPH radical scavenging and moderate antimicrobial activities of *M. baccifera* fruit extracts^13^. Effective utilization of *M. baccifera* fruits for human consumption has never been considered, and this is primarily due to lack of reliable scientific data on their nutritional potential. But young shoots (young tender culms) of various bamboos were extensively studied for their nutritional properties^14^,^15^. Here we report the nutritional properties of *M. baccifera* fruits, and our results could enhance the utility of this fruit as a food source/medicine. This study may also help to standardize and develop methods to mitigate the rodent outbreak and associated ecological problems during future flowering events.

## Results

### Amino acids, proteins

Free amino acids in *M. baccifera* fruit pericarp analyzed by HPLC revealed the presence of various essential (EA) and non-essential (N-EA) amino acids (Table 1). Lysine (EA, 397.67 μg/g, FW) and glutamic acid (N-EA, 367.00 μg/g, FW) were the two major amino acids in *M. baccifera* fruit pericarp. The free amino acid composition of fruit liquid at different growth stages is given in Table 2. Alanine (N-EA), histidine (EA), methionine (EA) and glycine (EA) were present in fruit liquid throughout the fruit maturation stages. Valine (EA), tyrosine (N-EA), leucine (EA) and isoleucine (EA) were detected only in the early stages of fruit liquid. Serine (N-EA) and glutamic acid (N-EA) were present only in the later stage of maturation i.e., after 28 days. Alanine (N-EA, 14.0 μg/mL), threonine (EA, 13.9 μg/mL), serine (N-EA, 10.8 μg/mL), glutamic acid (N-EA, 9.2 μg/mL), histidine (EA, 6.8 μg/mL) and phenyl alanine (EA, 6.2 μg/mL) were the major amino acids in mature (35 days) fruit liquids.

In Biuret protein assay, BSA standard only gave violet color and *M. baccifera* pericarps, seeds and fruit liquids did not give a similar color change or significant absorbances at 578 nm. This showed only very low or non-detectable levels of free protein contents. The average weight of precipitated (bound) protein from the fruits was also very low, seed 0.0040 g (0.08%, w/w) (n = 3) and pericarp 0.0069 g (0.14%, w/w) (n = 3), respectively. Dry almond (*Prunus dulcis*) seeds, known to be protein rich, were used as positive control and average weight of precipitated protein in them was found to be 14.84% (w/w) (n = 2).

### Sugars

**Figure 1:**
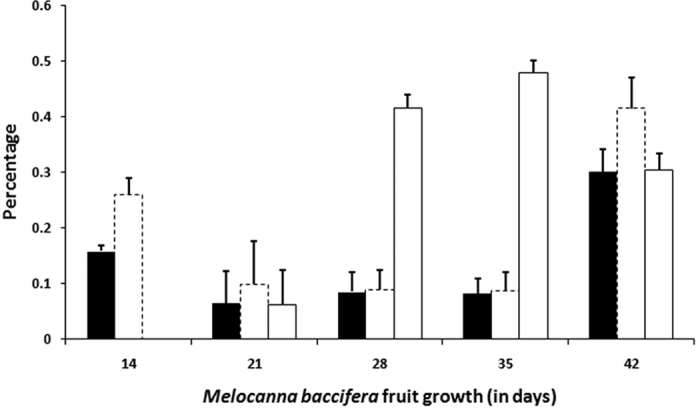
Sugars in the fruit liquid of *M. baccifera*. Filled bar - glucose; Dotted bar - fructose; Unfilled bar - sucrose. Fruit liquid was absent in the first week (7^th^ day); Inner seed of mature fruit was analyzed on 42^nd^ day; Each data point is an average of a minimum of 4 separate measurements on fruit samples.

**Figure 2:**
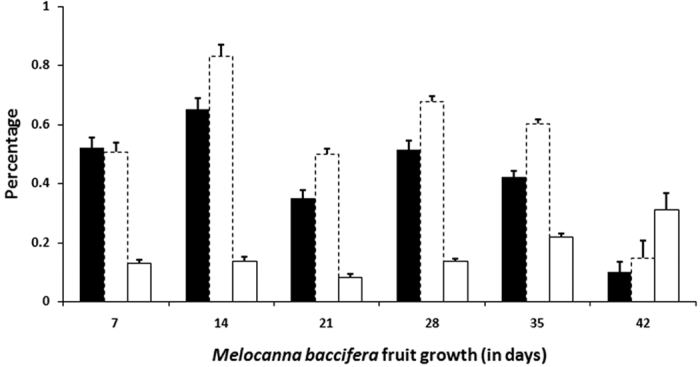
Sugars in the pericarp of *M. baccifera*. Filled bar - glucose; Dotted bar - fructose; Unfilled bar - sucrose. Each data point is an average of a minimum of 4 separate measurements on fruit samples.

Sugar analysis of *M. baccifera* on fruit liquid and pericarp showed a glucose-fructose-sucrose pattern that exists from pre-maturation to maturation stages (Figs. 1 and 2). In *M. baccifera* fruit pericarps, glucose-fructose-sucrose contents were 0.50, 0.51 and 0.13%, respectively, on the 7^th^ day of maturity. In fully matured fruit pericarps (42 days), their contents were 0.10, 0.15 and 0.31%, respectively (Fig. 2). In fruit liquids, glucose-fructose-sucrose contents were 0.16, 0.26 and 0.00% (on the 14^th^ day), 0.08, 0.09 and 0.48% (35^th^ day) and 0.30, 0.42 and 0.30% (42^nd^ day, seed instead of fruit liquid), respectively (Fig. 1). Fruit liquid was absent on the 7^th^ day (first week) of fruit growth.

### Total phenolics, phenolic acids

Total phenolic content in *M. baccifera* fruits estimated by Folin-Ciocalteau assay was 2.06 ± 0.003 mg ferulic acid equivalent/g, DW (n = 3). For identifying phenolic acids present in free, esterified and bound fractions of *M. baccifera* fruits, twelve most common phenolic acid standards (caffeic acid, cinnamic acid, chlorogenic acid, *p*-coumaric acid, gallic acid, gentisic acid, ferulic acid, salicylic acid, sinapic acid, syringic acid, vanillic acid, 4-hydroxy benzoic acid) were used as standards in HPTLC analysis. None of the standards (their R_f_ values) matched with the free fraction indicating absence or non-detectable levels of free phenolic acids in *M. baccifera* fruits. In esterified and bound fractions only ferulic acid (and no other phenolic acids) was detected. HPTLC quantification showed ferulic acid contents in esterified and bound fractions as 45.92 ± 6.86 μg/g, DW (n = 3) and 8.65 ± 0.36 mg/g, DW (n = 3), respectively.

### Lipids

Lipid analysis of *M. baccifera* fruits revealed the presence of fifteen saturated/unsaturated fatty acids (Table 3). *M. baccifera* fruits consisted mainly of saturated fatty acids with palmitic acid (C16:0) as the major constituent (0.13 g/100 g, DW (n = 3).

### Vitamins

Vitamin analysis of *M. baccifera* fruits showed the presence of vitamin B series, and neither vitamin C nor fat soluble vitamins (A,D,E,K) were detected in the fruits. Vitamin B3 and B2 dominated the profile with 2.68 and 2.13 mg/100 g, FW (n = 3), respectively (Table 4).

### Minerals

Mineral analysis by ICP-MS and AAS revealed *M. baccifera* fruit as rich in potassium, 831.5 mg/100 g, DW (n = 3) (Table 5).

### Fruit liquid, pericarp, seed and whole fruit feeding experiments

#### Fruit liquid drinking by rats

*M. baccifera* fruit liquid intake was 7.86 ± 1.73 mL/animal (n = 7) whereas water intake was only 2.55 ± 1.51 mL/animal (n = 7) during 6 h experiment. The average fruit liquid drinking affinity in rats was significantly higher (3.08 times) compared to intake of water (*p* < 0.0001).

#### Fruit liquid, sucrose, glucose and fructose feeding preference on rats

*M. baccifera* fruit liquid intakes by the rats in the three combinations were (fruit liquid: sucrose: water 2.27 ± 0.75: 5.07 ± 2.44: 3.40 ± 1.28 mL/animal (n = 4)), (fruit liquid: glucose: water 8.2 ± 0.5: 0.90 ± 0.56: 1.3 ± 0.9 mL/animal (n = 4)) and (fruit liquid: fructose: water 9.83 ± 0.58: 0.90 ± 0.53: 0.63 ± 0.32 mL/animal (n = 4)). Clearly, rats showed a high affinity to sucrose compared to the bamboo fruit liquid. But compared to glucose and fructose, the fruit liquid was consumed at significantly higher quantities (*p* < 0.0001). The average liquid intake per animal in 6 h in fruit liquid/sucrose/water, fruit liquid/glucose/water and fruit liquid/fructose/water combinations was 10.74, 10.40 and 11.36 mL, respectively.

#### Fruit pericarp, seed, normal food, water feeding preference on rats

Food intakes by rats in 24 h were (normal food: mature fruit pericarp: : water 9.69 ± 2.83: 9.99 ± 2.12 g: 19.97 ± 6.16 mL/animal, n = 6) and (normal food: mature seed: water 6.52 ± 1.27: 25.64 ± 6.86 g: 6.50 ± 2.96 mL/animal, n = 6). Rats showed a relatively higher preference to *M. baccifera* seed compared to its pericarp and normal food. Water intake is high for rats provided with fibrous fruit pericarp and considerably low when provided with seeds. Maximum weight loss of *M. baccifera* fresh fruit pericarp (50 g) and seed (50 g) in 24 h under similar conditions were 7.75 ± 1.97 g (n = 6) and 16.94 ± 3.15 g (n = 6), respectively. Normal food (50 g) under experimental conditions gained weight 0.75 ± 0.34 g (n = 6). Weight loss of water was minimal.

#### Fruit + normal food and fruit alone feeding experiments, serum hematological and biochemical parameters

Fruit feeding experiments were stopped on 8^th^ day since two animals from the group T II (fed with *M. baccifera* fruit alone) died. As shown in the Table 6, eight days consumption of *M. baccifera* fruit alone significantly reduced the body weights of animals in the T II group from 28.83 ± 1.42 g to 18.87 ± 1.43 g (*p* < 0.001, compared to control group) whereas control and T I group animals showed increments in their body weights from 26.16 ± 2.85 g and 27.33 ± 1.75 g to 28.46 ± 3.06 g and 30.36 ± 2.12 g, respectively. Serum protein content of the T II Group was significantly reduced to 1.03 ± 0.31 g/dl from 6.49 ± 0.69 g/dl of the control group (*p* < 0.001) and serum urea was significantly increased to 65.51 ± 10.3 mg/dl from 33.7 ± 6.01 mg/dl of control group (*p* < 0.001). In T II group, the hemoglobin content was reduced to 10.13 ± 1.14 g/dl from the control values of 12.28 ± 0.40 g/dl. Again, T II group animals showed significant increase in serum total cholesterol level (170.01 ± 14.71 mg/dl) than that of control animals (92.34 ± 14.70 mg/dl) (*p* < 0.001), with a remarkable reduction of HDL-cholesterol (33.93 ± 9.20 mg/dl) (Table 7). T I group showed a reduction in cholesterol (77.23 ± 3.05 mg/dl) with an HDL-cholesterol value of 48.42 ± 6.80 mg/dl. Serum triglyceride and alkaline phosphatase levels in all three groups did not show any significant changes. GOT and GPT values of T II group were significantly increased to 93.96 ± 9.10 IU and 35.05 ± 8.25 IU from 62.60 ± 9.34 IU and 25.78 ± 3.59 IU, respectively, of control group (*p* < 0.05). No significant changes were observed in GOT and GPT values between control and T I group (Table 7).

## Discussion

*M. baccifera* fruit pericarp and fruit liquid have essential and non-essential amino acids. Free amino acids are building blocks in protein synthesis and they also have critical roles in plant growth and physiology^16^. Lysine and glutamic acid are the two major amino acids found in *M. baccifera* fruits. Lysine is one of the essential amino acids required for growth and tissue repair in humans. It is also widely used as a nutritional supplement in foods, beverages, pharmaceuticals and animal feeds. Glutamic acid is a non-essential amino acid, and its derivatives and conjugates have been designed as effective anticancer agents^17^. A recent epidemiological study found that increase in dietary glutamic acid is likely to reduce blood pressure^18^. It also acts as a neurotransmitter in mammalian brain and as a precursor for the synthesis of γ-amino butyric acid (GABA) in GABAergic neurons^19^,^20^. Bamboo young shoots are also known to be rich in various essential and non-essential amino acids^14^,^15^. A preliminary study^8^ reported the protein content in *M. baccifera* fruits as 11.6%. Moreover, several popular scientific articles claimed high contents of protein in *M. baccifera* fruits, and inferred it as the major reason for rat multiplication during its flowering and fruiting^9^,^21^. In this study, by free and bound protein assays, we found only very low levels of proteins in *M. baccifera* fruit liquid, pericarp and seeds. This finding rejects the protein-driven rat multiplication hypothesis^9^,^21^ during fruiting.

*M. baccifera* fruit pericarp and fruit liquids at various growth stages showed glucose-fructose-sucrose patterns. Glucose and fructose showed gradual decrease in their levels with growth of the fruits, and sucrose content showed an increase with fruit growth. The low level of sucrose till 21 days could be attributed to sucrose metabolism into glucose and fructose, and its high content (after 21 days) could be associated with sucrose accumulation. Free sugars act as substrates for synthesis of complex carbohydrates such as starch and cellulose. Sugar signals are also critical in determining plant growth and development^22^,^23^. The sweetness and quality of fruits are also determined by sugars. As reported earlier^2^,^5^, we also observed various predators (rats, monkeys, borers etc.) visiting *M. baccifera* fruits. The presence of sugars may well be a critical factor attracting rodents and other predators into these fruits. Sugars, mainly sucrose, induce taste to the fruits and ultimately provide energy to animals and humans who consume them.

In feeding experiments, rats showed a clear affinity (3.08 times) to *M. baccifera* fruit liquid compared to water. They also showed high affinity to sucrose compared to the bamboo fruit liquid. But compared to glucose and fructose, fruit liquid was consumed at significantly higher quantities (*p* < 0.0001). The average liquid intake per animal in 6 h in fruit liquid/sucrose/water, fruit liquid/glucose/water and fruit liquid/fructose/water combinations was 10.74, 10.40 and 11.36 mL, respectively. The overall drinking of liquids went up when these combination of liquids were provided compared to water alone (2.55 mL). Rats, in feeding experiments, also showed a relatively higher preference to *M. baccifera* seed compared to pericarp and normal food. Water intake was high for rats provided with fibrous pericarp, and considerably low during seed intake. These data indicate that sucrose is the major taste factor in *M. baccifera* fruits and this explains the affinity of rats and other predators towards these fruits during gregarious flowering. Sugars are also making *M. baccifera* fruits an energy-rich diet to predators and act as one of the factors resulting in their (mainly rats) multiplication on fruit feeding.

Total phenolic content (*M. baccifera* fruits 2.06 ± 0.003 mg ferulic acid equivalent/g, DW) positively correlates with the antioxidant properties of a food source. Phenolics are secondary metabolites with at least one aromatic ring and one hydroxyl group. Based on the number of aromatic rings present they are classified either as simple phenols or polyphenols^24^. Phenolic acids are phenols with at least one carboxylic acid group. The role of plant phenolic acids in food industry has been associated with their nutritional and antioxidant properties. The quantity of phenolic acids present in plants as ‘free acids’ is very low as they are generally conjugated either to monosaccharides, polysaccharides, lignins or polyphenols through ester, ether or acetal bonds^24^. On HPTLC analysis, we detected only ferulic acid and no other common phenolic acids in *M. baccifera* fruits. HPTLC quantification showed ferulic acid contents in esterified and bound fractions as 45.92 ± 6.86 μg/g, DW and 8.65 ± 0.36 mg/g, DW, respectively. The content of bound ferulic acid in *M. baccifera* fruits is more than that of cereals (wheat, maize, rice etc.), which is reported to be in the range of 0.8 to 2.0 mg/g, DW^25^,^26^. Ferulic acid, known for several biological activities and thus reducing the risk of major diseases such as diabetes, heart disease, cancer etc., is a useful dietary component for human health^25^,^27^. Owing to its biological significance, several studies on its identification and estimation from various plants were reported in the literature^28^,^29^,^30^. The bound form of ferulic acid can be released and absorbed in the gastrointestinal tract through the actions of microorganisms, enzymes and glucose transporters^31^. Dietary intake of bound ferulic acid via whole grains has been proposed to prevent colon cancer and other digestive cancers^25^. Feruloylated oligosaccharides, obtained from insoluble feruloylated polysaccharides through the actions of hydrolytic enzymes without esterase activity, have numerous applications in food industry. Their antioxidant properties, better than free ferulic acid, inhibition against glycation and probiotic effects have been reviewed recently^32^. Similar studies on bound ferulic acid rich *M. baccifera* fruits may yield valuable information on the type and activity of feruloylated oligosaccharides.

*M. baccifera* fruit mainly consisted of saturated fatty acids with palmitic acid (C16:0) as the major constituent (0.13 g/100 g, DW). Palmitic acid is a good therapeutic agent with reported anticancer activities against human leukemic cell lines^33^. IC_50_ value of palmitic acid against leukemic cell lines has been reported in the range of 10–15 μg/ml^33^. *M. baccifera* fruits are rich source of palmitic acid, enhancing their biological significance. Vitamin B3 (2.68 mg/100 g, FW) and B2 (2.13 mg/100 g, FW) are the major vitamins in the fruits. Vitamin B3 (niacin) is an essential nutrient and an important pharmacological agent. It has been reported to lower serum lipid and cholesterol levels^34^,^35^. It also acts as a precursor for biosynthesis of NAD^+^, an important coenzyme which has profound physiological as well as neurological importance in humans^36^. Vitamin B2 (riboflavin) is needed for growth and maintenance of good health in humans. It helps to break down fats, carbohydrates and proteins by the body to release energy. Mineral analysis revealed *M. baccifera* fruit as rich in potassium (831.5 mg/100 g, DW). Bamboo shoots are also widely reported to be rich in potassium^37^. Potassium is a critical mineral essential for the proper functioning of cells, tissues and organs in human body. It is also important in maintaining fluid and electrolyte balance in the body. Potassium rich diet is attributed to protect against stroke-associated mortality^38^. Essential minerals like Mg (43.67 mg/100 g), Fe (9.14 mg/100 g), Cu (2.9 mg/100 g), Zn (3.27 mg/100 g) and Mn (1.17 mg/100 g) were also found in trace quantities in *M. baccifera* fruits.

In fruit feeding experiments, the weight reduction and significant changes in serum parameters of T II group animals are indications of semi-starvation and utilization of their stored energy in muscles, liver etc. Animals change from semi-starvation to starvation stage when they do not get sufficient energy from the food source and are forced to shift their physiology from carbohydrate dominated catabolism to lipid- or protein-dominated catabolism. This eventually results in severe hypoglycemia and leads to death of the animal. In T II group, two animals died in the afternoon of 8th day of the experiment. The dropped serum glucose level of this group (65.65 ± 4.82 mg/dl) (*p* < 0.05) indicates that the lethality could be due to hypoglycemia and other complications associated with starvation. In T II group, significant reduction in serum protein and increase in serum urea (*p* < 0.001) clearly show the starvation of animals due to less availability of food and a shift to protein catabolic phase with significant increase in blood urea level. The reduction in hemoglobin content may be due to severe protein catabolism observed in bamboo fruit alone fed animals. The reason that increased serum total cholesterol observed in T II group may be due to the primary shift of metabolism from a hexose dependent pathway to a lipid dependent one where the cholesterol stored in lipid droplets of adipose tissue is released to serum for obtaining energy^39^,^40^. Moreover, these feeding experiments showed that *M. baccifera* fruit alone is not sufficient for the maintenance of normal growth and physiology in animals. On the contrary, the normal food supplemented with bamboo fruit showed maintenance of body weight, serum biochemical and hematological parameters to normal values with a reduction in serum total cholesterol level. This is mimicking the bamboo field scenario where rodents take *M. baccifera* fruits along with other grains and food items. The actual mechanism of cholesterol lowering activity of *M. baccifera* fruits in normal fed animals may be attributed either to the presence of vitamin B3, whose cholesterol lowering activity has been widely reported^34^,^35^, or/and to other metabolites in the fruits.

The current belief is, *M. baccifera* suddenly dies after peak flowering/fruiting leaving the enhanced rat population short of fruits, and at this crunch they feed on nearby grain fields and destroy stored grains, leading to famine. But our results show a possible correlation between bamboo fruit production, ‘rat floods’ and subsequent famine. Bamboo flowering and fruit setting usually lasts for 2–5 years as observed by us^41^,^42^ and in other studies^2^,^5^. Fruit production gradually increases from minimal in first year to its peak in 2^nd^ or 3^rd^ year and then diminishes culminating in the death of these bamboos. Rats which devour the fruits in the initial years will have less bamboo fruits and hence consume more of normal foods (protein rich grains). Due to this mixed diet, rats multiply much faster resulting in more individuals. At the time of the peak fruit production (2–3 years) there will be more bamboo fruits and more rats. As a result, the rats due to the taste (of sugars) eat more bamboo fruits and for additional dietary requirements they attack nearby grain fields causing imminent famine. In the event of scarcity of normal food (grains such as rice, wheat), the rats are left with no option but to eat only bamboo fruits (low in protein, but tasty due to sugars) and this shift in food intake leads to starvation and ultimately to their death. Our study indicates this as one possible natural mechanism to check the rodent outbreak associated with *M. baccifera* flowering.

In conclusion, this study reports the nutritional properties of *M. baccifera* fruits for the first time. Free amino acids (lysine, glutamic acid), simple sugars (sucrose, glucose, fructose), phenolic acids (ferulic acid), fatty acids (palmitic acid), vitamins (B3) and minerals (potassium) identified in this unique bamboo fruit reveal its nutritional and therapeutic potentials. Significantly, the protein content (free, bound) in *M. baccifera* fruits is very low. *In vivo* rat feeding experiments showed that fruit alone is not a complete food, but along with other protein supplements, they are valuable food additives. This study enriches our knowledge of this unique bamboo fruit, which could lead to its better utilization during future flowering and fruiting events. Our results could also help in the successful management/prevention of rodent outbreaks and other ecological and social problems associated with fruiting of *M. baccifera*.

## Methods

### Chemicals, reagents

Amino acid standard kit, caffeic acid, cinnamic acid, chlorogenic acid, *p*-coumaric acid, gallic acid, gentisic acid, *trans*-ferulic acid, salicylic acid, sinapic acid, syringic acid, vanillic acid and 4-hydroxy benzoic acid were purchased from Sigma Aldrich, Bengaluru, India. β-Mercaptoethanol, PMSF, Tris-HCl, ammonium acetate, phenol, bovine serum albumin, glucose, fructose and sucrose were purchased from SRL, Mumbai, India. Serum glutamate pyruvate transaminase (GPT), serum glutamate oxaloacetate transaminase (GOT), alkaline phosphatase (ALP), serum urea, triglycerides, total cholesterol, high-density lipoprotein cholesterol and total protein were determined using commercially available kits (SPAN Diagnostics Ltd., Surat, India).

### Melocanna baccifera fruits

*M. baccifera* from various locations in north east India was introduced into the Bambusetum of Jawaharlal Nehru Tropical Botanic Garden and Research Institute (N 08°45. 262–430′ E 077°01. 429–583′) during 1988 to 1996 and its growth, multiplication and interaction with other habitats are under constant observation^43^. *M. baccifera* gregarious flowering during 2009 to 2015 produced clumps with attractive flowers and fruits (Fig. S1) and our studies were centered on five clumps (Accession numbers 58, 359, 365, 394, 403) in JNTBGRI Bambusetum^43^. Clump 58 was introduced from Forest Research Institute, Dehra Dun in 1988 (under cultivation) and clumps 359, 365, 394 and 403 were introduced from four locations (all wild) from Manipur in 1996. The altitude of origin varies from 435 m (Dehra Dun, clump 58) to 1061 m (Saikul, clump 365) above MSL^43^ (see Supplementary information). The altitude of JNTBGRI Bambusetum varies between is 79–186.6 m above MSL. *M. baccifera*, due to its adaptability to survive in different ecological habitats, is widely cultivated in many gardens around the globe.

In this study, *M. baccifera* fertilized flowers borne in the respective five clumps grown in JNTBGRI Bambusetum were marked and their progressive development from young fruits to mature ones at intervals of 7, 14, 21, 28, 35 and 42 days were carefully recorded in the field. Fruits at various growth stages were plucked and their size and fruit/seed weights were noted. Fruits were also cut open, fruit liquid contents (filled in cavities of 14–35 days old fruits) were pipetted out, specific gravities measured and in mature ones seeds (embryo + scutellum) were removed with scalpel and weighed. Fruit length: 3.02 ± 0.51 (7 days), 6.78 ± 0.88 (14), 7.80 ± 1.13 (21), 8.41 ± 0.72 (28), 9.11 ± 0.76 (35), 9.93 ± 1.51 cm (42 days) (n = 30). Fruit breadth: 0.51 ± 0.11 (7 days), 2.34 ± 0.71 (14), 3.10 ± 0.64 (21), 3.80 ± 0.66 (28), 4.87 ± 0.80 (35), 5.49 ± 0.62 cm (42 days) (n = 30). Fruit weight: 0.23 ± 0.12 (7 days), 11.09 ± 7.34 (14), 21.37 ± 10.63 (21), 36.38 ± 17.80 (28), 75.02 ± 32.37 (35), 104. 39 ± 26.96 g (42 days) (n = 30). Fruit liquid: 0.00 ± 0.00 (7 days), 0.53 ± 0.38 (14), 0.99 ± 0.52 (21), 1.19 ± 0.61(28), 0.95 ± 0.72 (35) (n = 30), 0.25 mL (42 days) (n = 1). Average specific gravities of fruit liquids were 1.00 ± 0.00 (n = 30), and did not show significant variations with growth stage of the fruit. Average mature fruit and seed weights were 104.39 ± 26.96 (42 days) (n = 30) and 6.09 ± 1.92 g (42 days) (n = 30), respectively. Average mature seed weight (42 days) was only 5.83% of total fruit wt. *M. baccifera* specimens were authenticated by Dr. K. C. Koshy, one of the authors, and voucher specimens were deposited at the Herbarium (TBGT) of the Institute (see Supplementary information).

### Amino acids

Amino acid composition of *M. baccifera* fruit pericarp was analyzed using HPLC-1200 Infinity series (Agilent Technologies, USA) equipped with a C18 column (250 mm × 4.6 mm, 5 μm), binary pump delivery system, robotic autosampler and diode array detector. *M. baccifera* fresh fruit pericarp (1 g) was homogenized and extracted (10 mL × 2; 15 min each) with 80% methanol, filtered, concentrated under reduced pressure. These pericarp samples or standard amino acids (20 μL) were automatically mixed with *O*-phthalaldehyde/β-mercaptoethanol reagent (50 μL) before injected onto the C18 column. The column was maintained at 40 °C and the detection wavelength was set at 340 nm. Mobile phase A: 0.05 M Na_2_HPO4 (pH 5.2):methanol:tetrahydrofuran (80:19:1), mobile phase B: 0.05 M Na_2_HPO4 (pH 5.2):methanol (20:80), flow rate of 1 mL/min. A linear gradient changing from 0% to 100% of mobile phase B over a time period of 52 min was used. Amino acids in pericarp were expressed as μg/g, FW (Table 1)^44^.

Amino acid compositions of *M. baccifera* fruit liquids at 14, 21, 28 and 35 days (of maturity) were analyzed using a HPLC-LC 10AS (Shimadzu, Japan) equipped with a Na type (ISC-07/S1504 Na, 19 cm × 5 mm) column packed with strongly acidic cation exchange resin (styrene divinyl benzene copolymer with sulphinic group) and a fluorescence detector^45^ (Table 2).

### Free proteins

*M. baccifera* fruit liquids (100 μL each) or fruit pericarp/seed (500 mg each, homogenized in 5 mL buffer, supernatant) (100 μL each) or BSA (protein standard) (10, 30, 50 μL; i. e., 130, 390, 650 μg) and Biuret reagent (1 mL) were mixed, total volume was made up to 1.5 mL with water and incubated at 37 °C for 5 min. After incubation, optical densities were recorded at 578 nm with water as a blank. BSA (protein standard) solution was prepared by mixing 100 μL BSA (6.5 g/dL) and 400 μL of water. Fruit liquid, pericarp and seed samples (at various growth stages or concentrations) were repeatedly tested.

### Bound proteins

Fresh *M. baccifera* fruit pericarp and seed (of three fruits) were cut into small pieces and 10 g each were ground using a laboratory grinder. Ground pericarp (5 g each) and seed (5 g each) were subjected to the phenol extraction protocol^46^. Briefly, *M. baccifera* pericarp (5 g) or seed (5 g) was ground in liquid nitrogen with glass powder and 75 mL extraction buffer (0.7 M sucrose, 0.1 M KCl, 0.5 M Tris-HCl, 50 mM EDTA, pH 7.5, 2% β-mercaptoethanol, 1 mM PMSF made in isopropanol). Into this mixture, 75 mL of phenol saturated with Tris-HCl, pH 7.5 was added, shaken at 4 °C (30 min), centrifuged at 4 °C for 30 min at 12,000 rpm and the upper phenol phase was collected. Equal volume of extraction buffer was added to the phenol phase, stirred for 30 min at 4 °C and centrifuged for 30 min at 12,000 rpm at 4 °C. Phenol phase was again collected and extraction/centrifugation was repeated for three times. Final upper phenol phase (about 50 mL) was collected in a conical flask, 5 times volume of 0.1 M ammonium acetate (made in methanol) was added, shaken well and kept at −20 °C overnight. Proteins in *M. baccifera* pericarp or seed were precipitated by this method. After centrifugation steps with methanol and acetone, the pellets were dried and weighed^46^. Dry almond (*Prunus dulcis*) seeds, known to be protein rich, were used as positive control and protein in almond seeds were precipitated, dried and weighed as in the same protocol (n = 2).

### Sugars

Sugars in *M. baccifera* fruits were analyzed by HPTLC-densitometry (Camag, Switzerland) using pre-coated silica gel 60 F254, 20 × 10 cm, 0.2 mm thickness (E. Merck, Germany). The silica gel plates were pretreated by dipping in 0.2 M aqueous solution of monobasic potassium phosphate and wet plates were dried at 90 °C for 45 min. Plates were then cooled at room temperature for 2 h and stored in a desiccator^47^. *M. baccifera* pericarp and fruit liquid samples at various growth stages were applied onto these pretreated silica gel plates as 6 mm wide bands with automatic Linomat V sample applicator (Camag, Switzerland), fitted with a micro syringe, in N_2_ flow (application rate 100 nL/s, space between two bands 13.0 mm, slit dimension 6 × 0.4 mm, scanning speed 20 mm/s). The plates were developed three times, each time to a distance of 80 mm, with acetonitrile-water (85:15, v/v) in a paper-lined twin-trough HPTLC chamber (Camag, Switzerland), which was equilibrated with the mobile phase for 10 min. Fresh solvent was used for each run and between runs the plate was thoroughly dried with a hair drier^48^. After the third development the plate was dried, derivatized by spraying with anisaldehyde-sulphuric acid reagent and heated at 110 °C for 10 min. The plates were scanned densitometrically at 580 nm (tungsten lamp) using TLC Scanner 3 (Camag, Switzerland) equipped with winCATS software. *M. baccifera* fruit liquid or pericarp or seed at various growth stages were taken and each individual growth stage (data point) was generated by a minimum of 4 sample measurements (Figs 1 and 2). Fruit liquid (14, 21, 28, 35 days; 0.5 μL each) was directly loaded on to the plates. Fruit pericarp/seed (7, 14, 21, 28, 35 days and inner seed of mature fruit, 42 days; 0.5 g each) was extracted twice with water (10 mL × 2; 30 min each), filtered, concentrated under reduced pressure to 10 mL and 5 μL (each) was loaded. Sucrose, glucose and fructose stock solutions (1.0 μg/μL) were made in water and spotted in the range (sucrose 0.1, 0.2, 0.3, 0.4, 0.5, 0.6, 0.7, 0.8 μg), (glucose 0.1, 0.2, 0.3, 0.4, 0.6, 0.8, 1.0 μg), (fructose 0.1, 0.2, 0.3, 0.4, 0.6, 0.8, 1.0 μg) to obtain their calibration graphs. Sucrose (y = 6336x + 10.60, R^2^ = 0.997; R_f_ = 0.19 ± 0.01, n = 30), glucose (y = 5112x + 5.320, R^2^ = 0.994; R_f_ = 0.29 ± 0.02, n = 30), fructose (y = 5443.x + 44.56, R^2^ = 0.999; R_f_ = 0.35 ± 0.02, n = 30).

### Total phenolics

Total phenolic content in *M. baccifera* fruits was estimated using Folin-Ciocalteau assay^49^. Dried fruit powder (1 g) was extracted with 10 mL of 80% aqueous methanol containing 1% HCl for 2 h at room temperature using a magnetic stirrer. The mixture was centrifuged at 2000 rpm for 15 min and the supernatant was collected. The pellets were washed (80% aqueous methanol) and supernatants were pooled. Volume of the supernatant was reduced to 5 mL under reduced pressure at 40 °C and used for the assay. Either 100 μL of extract or various known concentrations of standard ferulic acid was made up to 1 mL with water and mixed with 4 mL Folin-Ciocalteau reagent (previously diluted to 1:10 with water). Mixtures were allowed to stand for 5 min at room temperature, then 4 mL of 20% Na_2_CO_3_ was added and again kept at room temperature for 15 min. Absorbances were measured at 765 nm and the concentrations were expressed as mg ferulic acid equivalent per gram, DW. All experiments were carried out three times with different *M. baccifera* fruits.

### Extraction of soluble, insoluble phenolic acids

Free, esterified and bound phenolic acids in *M. baccifera* fruits were analyzed as described in the literature^50^ with modifications. Dried defatted fruit powder (5 g) was cold extracted with methanol:acetone:water (7:7:6) (50 mL × 3; 60 min each), filtered, concentrated under reduced pressure, acidified to pH ~ 2 (using 6N HCl) and centrifuged. Aqueous supernatant thus obtained was first extracted twice with hexane to remove low polar molecules, and then extracted 4 times with diethyl ether-ethyl acetate (1:1) to obtain free phenolic acid fraction. The remaining aqueous phase, containing soluble phenolic acid esters, was concentrated under reduced pressure and hydrolyzed (for identifying the phenolic moiety) with 25 mL of 4N NaOH for 4 h at room temperature. The hydrolysate was acidified with 6N HCl and extracted 4 times with diethyl ether-ethyl acetate (1:1). The organic fraction was concentrated under reduced pressure and marked as phenolic acid ester fraction. The residue, containing bound insoluble phenolic acids, remained after methanol:acetone:water extraction was alkaline hydrolyzed under the similar conditions to release and extract phenolic acid moieties from their bound forms.

### Identification, estimation of phenolic acids

Three phenolic acid fractions and authentic phenolic acid standards (caffeic acid, cinnamic acid, chlorogenic acid, *p*-coumaric acid, gallic acid, gentisic acid, *trans*-ferulic acid, salicylic acid, sinapic acid, syringic acid, vanillic acid, 4-hydroxy benzoic acid) were either dissolved in methanol or in chloroform-methanol (1:1) and applied by Linomat V sample applicator of HPTLC (Camag, Switzerland) onto a pre-coated silica gel TLC plate (60 F254, E. Merck, Germany, 20 × 10 cm, 0.2 mm thickness). The plate was developed up to 80 mm in 10 mL of chloroform:methanol (8.5:1.5) mobile phase in a twin trough glass chamber (Camag, Switzerland) under saturated conditions. After development, the plate was documented under UV 254 and 366 nm using Reprostar 3 (Camag, Switzerland) and then scanned densitometrically at 254 nm using TLC Scanner 3 (absorbance mode) (Camag, Switzerland) for estimation of phenolic acids. Percentage phenolic acid (ferulic acid) contents (% ± SD, n = 3, DW) were calculated from peak areas using the standard curve (y = 13096x + 578.8, R^2^ = 0.988) with a linear relationship in the range 0.04 to 0.8 μg.

### Lipids

*M. baccifera* mature fruit was dried, powdered and 30 g fruit powder was subjected to Soxhlet extraction with n-hexane (300 mL) for 8 h, extract was concentrated to get the crude lipid fraction (0.06 g, 0.2%). Crude lipids in the extracted fraction were converted to their methyl esters^51^. Briefly, the crude lipid fraction was refluxed at 70 °C for 4 h with 2% sulfuric acid in methanol, cooled to room temperature and then the esters were extracted into ethyl acetate. This fraction was further passed over anhydrous sodium sulfate and concentrated using a rotary evaporator to obtain the fatty acid methyl esters (FAME). Fatty acid composition of the FAME was analyzed using a 6890 N series Gas Chromatograph (Agilent Technologies, USA) equipped with a Flame Ionization Detector (FID) with a split injector. A fused silica capillary column (DB-225, 30 × 0.32 mm i.d., J&W Scientific, USA) was used with the injector and detector temperatures maintained at 230 and 250 °C, respectively. Oven temperature was programmed at 160 °C for 2 min and then raised at a rate of 4 °C/min to 230 °C, and held for 20 min at 230 °C. Nitrogen gas was used as carrier gas at a flow rate of 1.5 mL/min. Structures of the fatty acid methyl esters were analysed using a 6890 N GC-MS Series (Agilent Technologies, USA) equipped with a DB-225 column (30 m × 0.25 mm i.d.) connected to a 5973 Mass Spectrometer operating in the EI mode (70 eV; m/z 50–550; source temperature 230 °C; quadruple temperature 150 °C). Column temperature was initially maintained at 100 °C for 2 min, increased to 300 °C at 10 °C/min with a hold time of 20 min at 300 °C. Inlet temperature was maintained at 300 °C and split ratio was 50:1. Structural assignments were performed based on the interpretation of mass spectrometric fragmentation with that of standard FAME mixture and also matching with the library (Table 3). Three separate *M. baccifera* fruit samples were analyzed for their fatty acid contents.

### Vitamins

Vitamin content in *M. baccifera* whole fruit was analyzed using standard protocols (Table 4). Vitamin B1, B2, B3, B5 and B6 were analyzed following a HPLC method^52^. Vitamin B7, B9 and B12 were quantified using enzyme linked immunosorbent assay according to the manufacturer’s instruction (RIDASCREEN^®^ developed by R-Biopharm^®^ Darmstadt, Germany). Vitamin C was measured by 2,6-dichlorophenol indophenol method^53^. Fat soluble vitamins were measured using the methods described elsewhere^54^,^55^.

### Minerals

*M. baccifera* dried fruit powder was subjected to microwave-assisted digestion before carrying out mineral analysis (Table 5). Dried fruit powder (0.1 g) was treated with mixture of 7 mL nitric acid (Suprapur grade, Merck, Germany) and 1 mL H_2_O_2_ in a Multiwave 3000 Microwave Digestion System (Anton Paar, Graz, Austria). Microwave digestion, operation conditions: (i) 350 watts with ramp time of 0 min, hold time of 25 min, (ii) 500 watts with ramp time of 5 min, hold time of 35 min. The digested solution was made to 100 mL with high purity water (Barnstead Smart2Pure, Thermo Scientific, Germany). An inductively coupled Plasma Spectrometer iCAP Qc (Thermo Scientific, Germany) equipped with a quadrupole mass analyser and an autosampler ASX-520 (CETAC, Omaha, NE, USA) was used for trace mineral analysis. Data acquisition was carried out using Qtegra software. Operation conditions: spray chamber temperature 2.70 °C, peristaltic pump speed 40 RPM, cool flow 14.00 L/min, sampling depth 5, plasma power 1550 W, axillary flow 0.80 L/min, nebulizer flow 0.97 L/min and number of main runs 3.

Major minerals, Na, K and Ca, were analyzed using a PinAAcle 900H Atomic Absorption Spectrometer (Perkin-Elmer, Singapore) with flame and graphite furnace. *M. baccifera* dried fruit powder (2 g) was ashed at 500 °C, dissolved in HCl, made up to 10 mL with distilled water and aspirated in AAS^56^ (Table 5).

### Fruit liquid, pericarp, seed, fruit feeding experiments

#### Animals

Inbred male and female Wistar rats and male Swiss albino mice reared in JNTBGRI Animal House caged in uniform hygienic conditions and fed with standard pellet diet (Kamadhenu Feeds, Bangalore) and water *ad libitum* were used for experiments. The animals were maintained a 12 h light/dark cycle, with a temperature range of 23–25 °C and a relative humidity of 45–55% throughout the experimental period. Animal studies were carried out in accordance with the guidelines of Committee for the Purpose of Control and Supervision of Experiments on Animals (CPCSEA), Government of India. These animal experiments were also approved (B-01/12/2011/PPD-03 dated 19.12.2011) by the Institute Animal Ethics Committee (IAEC).

#### Fruit liquid drinking preference by rats

Fourteen normal female Wistar rats (178–200 g) were divided into two groups of seven each. Group I and II were kept as control and test, respectively, and animals were caged individually. Control group (I) animals were provided with 10 mL of drinking water (per animal) and the test group (II) animals were provided with 10 mL of *M. baccifera* fruit liquid (of 21–28 days old fruits) (per animal) for 6 h using feeding bottles under similar conditions. Animals were fed with standard food pellets during the experiment. After 6 h, the drinking rates by control and test group animals were measured.

#### Fruit liquid, sucrose, glucose, fructose feeding preference by rats

Twelve normal male Wistar rats (229–249 g) were divided into three groups of four animals each. Animals in each group were individually caged and fed with standard food pellets. Animals in group I were individually provided with 10 mL fruit liquid (of 21–28 days old fruits), 10 mL drinking water and 10 mL of 0.5% sucrose. Group II animals were (individually) provided with 10 mL fruit liquid (of 21–28 days old fruits), 10 mL drinking water and 10 mL of 0.5% glucose. Animals in Group III were (individually) provided with 10 mL fruit liquid (of 21–28 days old fruits), 10 mL drinking water and 10 mL of 0.5% fructose. To animals (in each group), the liquids were provided with three separate feeding bottles simultaneously. The amounts of liquids consumed by all three groups were measured after 6 h.

#### Fruit pericarp, seed, normal food, water feeding preference by rats

Twelve normal male Wistar rats (184–233 g) were divided into two groups of six animals each and the animals in each group were caged individually. Group I animals were separately supplied with 50 g of normal food pellet and 50 g of fresh *M. baccifera* pericarp (of mature fruit, 42 days). Group II animals were separately supplied with 50 g of normal food pellet and 50 g of fresh *M. baccifera* seed (of mature fruit, 42 days). All animals were provided with 200 mL drinking water. The pericarp and seed were chopped finely and in an identical manner for easier accessibility to the animals. After 24 h, the intake of normal food, fruit pericarp, seed and water were measured in each cage to determine the feeding preferences by the rats. To find out the weight loss (due to water loss) during the experimental hours, 50 g each of fresh *M. baccifera* pericarp, seeds and normal food pellets were kept separately for 24 h in the Animal House and their weight losses were determined (n = 6, each). Loss of water under similar experimental conditions were also verified (n = 6).

#### Fruit + normal food and fruit alone feeding experiments, serum hematological and biochemical parameters

Eighteen Swiss albino male mice (23–30 g) were selected and divided into three groups of six animals each. Control animals were provided with 50 g of standard food pellet, the first test group (T I) animals received 50 g of standard food pellet along with 50 g of fresh finely chopped *M. baccifera* fruits (mature fruits) and the second test group (T II) of animals received 50 g of fresh finely chopped *M. baccifera* fruits alone (mature fruits), every day in an identical manner. All three groups were provided with 100 mL water daily. The animal weights were taken at the start and end of these experiments (Table 6). These experiments were designed for 14 days, but stopped on 8^th^ day since two animals in group T II died. So on the 8th day of the experiment, the blood samples from tail vein of all animals were collected to measure the hemoglobin contents, and then animals were sacrificed, blood samples were collected by cardiac puncture and the serum biochemical parameters were determined (glucose, urea, GOT, GPT, total cholesterol^57^, HDL cholesterol^58^, triglycerides^59^ and alkaline phosphatase^60^) using commercial assay kits (Table 7).

#### Statistics

Results of various assays are expressed as mean ± SD Statistical comparisons were done using one way ANOVA followed by Dunnett’s Post-hoc comparison. Unpaired Student’s *t*-test was used when only two groups were involved. Values of *p* < 0.05 were considered as statistically significant.

## Additional Information

**How to cite this article**: Govindan, B. *et al.* Nutritional properties of the largest bamboo fruit *Melocanna baccifera* and its ecological significance. *Sci. Rep.* **6**, 26135; doi: 10.1038/srep26135 (2016).

## Supplementary Material

Supplementary Information

## Figures and Tables

**Table 1:** Amino acid composition of *M. baccifera* fruit pericarp.

Amino acid	μg/g, FW
Alanine	58.67 ± 14.29
Arginine	152.33 ± 17.79
Aspartic acid	43.33 ± 8.33
Glutamic acid	367.00 ± 38.11
Glycine	60.33 ± 25.79
Histidine	192.33 ± 45.62
Isoleucine	2.20 ± 1.39
Leucine	1.57 ± 0.75
Lysine	397.67 ± 137.88
Methionine	8.33 ± 1.15
Phenylalanine	10.33 ± 2.52
Serine	60.33 ± 15.37
Threonine	19.00 ± 10.58
Tryptophan	27.00 ± 6.93
Tyrosine	3.00 ± 1.00
Valine	2.33 ± 0.58

Data are expressed as mean ± SD of three independent samples.

**Table 2:** Amino acid compositions of *M. baccifera* fruit liquids at various growth stages.

Amino acid	Fruit liquids at various growth stages, in days (μg/mL)			
	14	21	28	35
Alanine	6.5	7.2	4.3	14.0
Aspartic acid	12.1	12.8	−	3.2
Glutamic acid	−	−	7.6	9.2
Glycine	0.9	1.2	0.4	0.4
Histidine	1.0	1.8	2.4	6.8
Isoleucine	2.6	4.1	−	−
Leucine	2.4	1.3	−	−
Methionine	1.3	0.1	2.1	2.4
Phenyl alanine	0.3	0.6	−	6.2
Serine	−	−	12.5	10.8
Threonine	5.8	−	−	13.9
Tyrosine	0.3	0.5	−	−
Valine	5.7	4.3	−	−

**Table 3:** Fatty acid composition of *M. baccifera* fruit analyzed by GC and GC-MS.

Lipid number	g/100 g, DW
C12:0	0.0012 ± 0.0010
C14:0	0.0016 ± 0.0014
C14:1	0.0024 ± 0.0011
C15:0	0.0067 ± 0.0033
C16:0	0.1324 ± 0.0239
C17:0	0.0013 ± 0.0014
C18:0	0.0075 ± 0.0008
C18:1	0.0085 ± 0.0020
C18:2	0.0046 ± 0.0046
C20:0	0.0019 ± 0.0008
C20:1	0.0012 ± 0.0010
C20:2	0.0078 ± 0.0059
C22:0	0.0024 ± 0.0013
C23:0	0.0056 ± 0.0054
C24:0	0.01003 ± 0.0101

Data are expressed as mean ± SD of three independent samples.

**Table 4:** Vitamin analysis of *M. baccifera* fruits.

Vitamin	mg/100 g, FW
B1	0.785 ± 0.021
B3	2.675 ± 0.346
B5	2.125 ± 1.959
B7	0.023 ± 0.001
B9	0.007 ± 0.005
B12	0.001 ± 0.001

Data are expressed as mean ± SD of three independent samples.

**Table 5:** Mineral analysis of *M. baccifera* fruits by ICP-MS and AAS.

Mineral	mg/100 g, DW
Ag	ND
Al	43.89 ± 20.52
As	ND
Ba	0.49 ± 0.21
Ca	2.01 ± 1.07
Cd	0.06 ± 0.05
Co	0.10 ± 0.14
Cr	1.07 ± 1.34
Cs	ND
Cu	2.90 ± 0.47
Fe	9.14 ± 1.23
In	ND
K	831.50 ± 140.60
Li	0.12 ± 0.08
Mg	43.68 ± 6.88
Mn	1.17 ± 0.66
Na	6.75 ± 0.95
Ni	0.07 ± 0.02
Pb	0.14 ± 0.03
Rb	11.22 ± 2.15
Se	ND
Sr	0.63 ± 0.39
Zn	3.27 ± 0.50

Data are expressed as mean ± SD of three independent samples.

**Table 6:** Effect of *M. baccifera* fruit on body weight of male mice (fed for eight days).

Groups	1st day(Weight, g)	5th day(Weight, g)	8th day(Weight, g)
Control	26.16 ± 2.85	27.83 ± 3.06	28.46 ± 3.06 (+2.3)
T I	27.33 ± 1.75	29.18 ± 1.98	30.36 ± 2.12 (+3.03)
T II	28.83 ± 1.42	20.30 ± 1.28*	18.87 ± 1.43** (−9.95)

(Values are mean ± SD n = 6 (except T-II group, where n = 4); *p < 0.05, **p < 0.001 (compared to control); Difference in body weights were given in bracket; On 8th day, in bamboo fruit fed group, 2 animals died hence n = 4 in that group. All animals were sacrificed on 8^th^ day and blood samples were collected).

**Table 7:** Effect of *M. baccifera* fruit on hematological and serum biochemical parameters of male mice.

Groups	Control	T I	T II
Protein (g/dl)	6.49 ± 0.69	5.97 ± 0.66	1.03 ± 0.31**
Glucose (mg/dl)	102.16 ± 5.82	107.86 ± 6.40	65.65 ± 4.82*
Urea (mg/ dl)	33.7 ± 6.01	34.4 ± 3.41	65.51 ± 10.30**
GOT (IU/l)	62.60 ± 9.34	61.29 ± 9.40	93.96 ± 9.10*
GPT (IU/l)	25.78 ± 3.59	23.03 ± 3.55	35.05 ± 8.25*
Total cholesterol (mg/dl)	92.34 ± 14.70	77.23 ± 3.05	170.01 ± 14.71**
HDL cholesterol (mg/dl)	49.63 ± 4.94	48.42 ± 6.80	33.93 ± 9.20*
Triglycerides (mg/dl)	68.25 ± 8.14	72.33 ± 9.07	64 ± 6.80
ALP (KAU)	8.09 ± 0.68	7.13 ± 1.8	9.08 ± 0.62
Hb (g/dl)	12.28 ± 0.402	12.67 ± 1.46	10.13 ± 1.14

(Values are mean ± SD; n = 6 (except T II group, where n = 4); *p < 0.05, **p < 0.001 (compared to control group); GPT, Glutamate pyruvate transferase; GOT, Glutamate oxaloacetate transaminase; AP, Alkaline phosphatase; On 8^th^ day, in T II group, 2 animals died and hence all animals were sacrificed on 8^th^ day).

## References

[b1] McClure F. A. The Bamboos: A Fresh Perspective (Harvard University Press, Cambridge, 1966).

[b2] Banik R. L. Biology and Silviculture of Muli Bamboo Melocanna baccifera (Roxb.) Kurz., 1-237 (Department of Science and Technology, National Mission on Bamboo Applications (NMBA), TIFAC, New Delhi, 2010).

[b3] Jeeva S., Kiruba S., Lalthruatluanga H., Prasad M. N. V. & Rao R. R. Flowering of *Melocanna baccifera* (Bambusaceae) in northeastern India. Curr. Sci. 96, 1165–1166 (2009).

[b4] Majumdar K., Nath A. J., Gupta A. K. & Datta B. K. Bamboo invasion: threat to primate conservation in North East India. Curr. Sci. 108, 1969–1971 (2015).

[b5] Janzen D. Why bamboos wait so long to flower. Annu. Rev. Ecol. Syst. 7, 347–391 (1976).

[b6] Aplin K. & Lalsiamliana J. Chronicle and impacts of the 2005-09 mautam in Mizoram. In, Rodent Outbreaks: Ecology and Impacts (eds Singleton G. R., Belmain S. R., Brown P. R. & Hardy B.) Ch. 1, 13–47 (International Rice Research Institute, 2010).

[b7] Gadgil M. & Prasad S. Ecological determinants of life history evolution of two Indian bamboo species. Biotropica 16, 161 (1984).

[b8] Rokhuma C. Tam do pawl in engnge a tih? (The secret of famines found.) The Activities of the Anti-famine Campaign Organisation Mizoram on Mautam Famine 1959 (Gosen Press, Aizawl, 1988).

[b9] Normile D. Holding Back a Torrent of Rats. Science 327, 806–807 (2010). 20150483 10.1126/science.327.5967.806

[b10] Wilkins A. Massive plagues of rats swarm across India every fifty years. http://io9.com/5694107/massive-plagues-of-rats-swarm-across-india-every-fifty-years, (2010) (18/02/2016).

[b11] Bhardwaj S. & Gakhar S. K. Ethnomedicinal plants used by the tribals of Mizoram to cure cuts & wounds. Indian J. Tradit. Know. 4, 75–80 (2005).

[b12] Kuddus *et al.* Secondary metabolites from *Melocanna baccifera* (Roxb.). Asian J. Chem. 23, 85–88 (2011).

[b13] Kuddus M. R., Rumi F., Haque M. R., Hassan M. A. & Rashid M. A. Assessment of antioxidant, antimicrobial and cytotoxic properties of fruits of *Melocanna baccifera* (Roxb.) Kurz. *Turk*. J. Pharm. Sci. 10, 185–192 (2013).

[b14] Chongtham N., Bisht M. S. & Haorongbam S. Nutritional properties of bamboo shoots: potential and prospects for utilization as a health food. Compr. Rev. Food Sci. Food Saf. 10, 153–168 (2011).

[b15] Nongdam P. & Tikendra L. The nutritional facts of bamboo shoots and their usage as important traditional foods of Northeast India. Int. Sch. Res. Notices 2014, 679073 (2014). 10.1155/2014/679073 PMC4897250 27433496

[b16] D’Mello J. P. F. Amino Acids in Higher Plants (CABI International, Oxfordshire, 2015).

[b17] Ali I., Wani W. A., Haque A. & Saleem K. Glutamic acid and its derivatives: candidates for rational design of anticancer drugs. Future Med. Chem. 5, 961–978 (2013). 23682571 10.4155/fmc.13.62

[b18] Stamler J. *et al.* Glutamic Acid, the main dietary amino acid, and blood pressure the INTERMAP Study (International collaborative study of macronutrients, micronutrients and blood pressure). Circulation 120, 221–228 (2009). 19581495 10.1161/CIRCULATIONAHA.108.839241 PMC4048930

[b19] Rindi G. & Ferrari G. The γ-aminobutyric acid and glutamic acid content of brains of rats treated with toxopyrimidine. Nature 183, 608–609 (1959). 13632808 10.1038/183608a0

[b20] Fagg G. E. & Foster A. C. Amino acid neurotransmitters and their pathways in the mammalian central nervous system. Neuroscience 9, 701–719 (1983). 6137788 10.1016/0306-4522(83)90263-4

[b21] Shoumatoff A. Waiting for the plague. http://www.vanityfair.com/news/2007/12/famine200712, (2007) (18/02/2016).

[b22] Smeekens S. Sugar-induced signal transduction in plants. Annu. Rev. Plant Biol. 51, 49–81 (2000). 10.1146/annurev.arplant.51.1.49 15012186

[b23] Lastdrager J., Hanson J. & Smeekens S. Sugar signals and the control of plant growth and development. J. Exp. Bot. 65, 799–807 (2014). 24453229 10.1093/jxb/ert474

[b24] Robbins R. J. Phenolic acids in foods: an overview of analytical methodology. J. Agric. Food. Chem. 51, 2866–2887 (2003). 12720366 10.1021/jf026182t

[b25] Adom K. K. & Liu R. H. Antioxidant activity of grains. J. Agric. Food. Chem. 50, 6182–6187 (2002). 12358499 10.1021/jf0205099

[b26] Sosulski F., Krygier K. & Hogge L. Free, esterified, and insoluble-bound phenolic acids. 3. Composition of phenolic acids in cereal and potato flours. J. Agric. Food. Chem. 30, 337–340 (1982).

[b27] Baby S. *et al.* UV induced visual cues in grasses. Sci. Rep. 3, 2738 (2013). 24061408 10.1038/srep02738 PMC3781395

[b28] Donno D. *et al.* Analytical fingerprint and chemometrics as phytochemical composition control tools in food supplement analysis: characterization of raspberry bud preparations of different cultivars. J. Sci. Food Agric. doi: 10.1002/jsfa.7494 (2015). 26459916

[b29] Peev C. I., Vlase L., Antal D. S., Dehelean C. A. & Szabadai Z. Determination of some polyphenolic compounds in buds of *Alnus* and *Corylus* species by HPLC. Chem. Nat. Compd. 43, 259–262 (2007).

[b30] Donno D. *et al.* Phytochemical fingerprint and chemometrics for natural food preparation pattern recognition: an innovative technique in food supplement quality control. J Food Sci. Technol. 1–13 doi: 10.1007/s13197-015-2115-6 (2015). 27162387 PMC4837732

[b31] Acosta-Estrada B. A., Gutiérrez-Uribe J. A. & Serna-Saldívar S. O. Bound phenolics in foods, a review. Food Chem. 152, 46–55 (2014). 24444905 10.1016/j.foodchem.2013.11.093

[b32] Ou J. & Sun Z. Feruloylated oligosaccharides: Structure, metabolism and function. J. Funct. Foods 7, 90–100 (2014).

[b33] Harada H. *et al.* Antitumor activity of palmitic acid found as a selective cytotoxic substance in a marine red alga. Anticancer Res. 22, 2587–2590 (2002). 12529968

[b34] Capuzzi D. M., Morgan J. M., Brusco O. A. & Intenzo C. M. Niacin dosing: relationship to benefits and adverse effects. Curr. Atheroscler. Rep. 2, 64–71 (2000). 11122726 10.1007/s11883-000-0096-y

[b35] Schachter M. Strategies for modifying high-density lipoprotein cholesterol: a role for nicotinic acid. Cardiovasc. Drugs Ther. 19, 415–422 (2005). 16453091 10.1007/s10557-005-5685-0

[b36] Sauve A. A. NAD+ and vitamin B3: from metabolism to therapies. J. Pharmacol. Exp. Ther. 324, 883–893 (2008). 18165311 10.1124/jpet.107.120758

[b37] Choudhury D., Sahu J. K. & Sharma G. D. Value addition to bamboo shoots: a review. J. Food Sci. Technol. 49, 407–414 (2012). 23904649 10.1007/s13197-011-0379-z PMC3550903

[b38] Khaw K. T. & Barrett-Connor E. Dietary potassium and stroke-associated mortality. A 12-year prospective population study. N. Engl. J. Med. 316, 235–240 (1987). 3796701 10.1056/NEJM198701293160502

[b39] Swaner J. C. & Connor W. E. Hypercholesterolemia of total starvation: its mechanism via tissue mobilization of cholesterol. Am. J. Physiol. 229, 365–369 (1975). 169705 10.1152/ajplegacy.1975.229.2.365

[b40] Sävendahl L. & Underwood L. E. Fasting increases serum total cholesterol, LDL cholesterol and apolipoprotein B in healthy, nonobese humans. J. Nutr. 129, 2005–2008 (1999). 10539776 10.1093/jn/129.11.2005

[b41] Koshy K. C. & Harikumar D. Reproductive biology of *Ochlandra scriptoria*, an endemic reed bamboo of the Western Ghats, India. Bamboo Science & Culture 15, 1–7 (2001).

[b42] Koshy K. C. & Mathew P. J. Does *Ochlandra scriptoria* flower annually or once in a lifetime? Curr. Sci. 96, 769–770 (2009).

[b43] Koshy K. C. Bamboos at TBGRI. (Tropical Botanic Garden and Research Institute, Thiruvananthapuram, 2010).

[b44] Antoine F. R., Wei C. I., Littell R. C. & Marshall M. R. HPLC method for analysis of free amino acids in fish using o-phthaldialdehyde precolumn derivatization. J. Agric. Food. Chem. 47, 5100–5107 (1999). 10606579 10.1021/jf990032+

[b45] Ishida Y., Fujita T. & Asai K. New detection and separation method for amino acids by high-performance liquid chromatography. J. Chromatogr. A 204, 143–148 (1981). 10.1016/s0021-9673(00)81650-7 7217249

[b46] Isaacson T. *et al.* Sample extraction techniques for enhanced proteomic analysis of plant tissues. Nat. Protoc. 1, 769–774 (2006). 17406306 10.1038/nprot.2006.102

[b47] Lee K. Y., Nurok D. & Zlatkis A. Determination of glucose, fructose and sucrose in molasses by high-performance thin-layer chromatography. J. Chromatogr. A 174, 187–193 (1979). 10.1016/s0021-9673(00)89984-7 721939

[b48] Sherma J. & Zulick D. L. Determination of fructose, glucose, and sucrose in beverages by high-performance thin layer chromatography. Acta Chromatogr. 6, 7–12 (1996).

[b49] Velioglu Y. S., Mazza G., Gao L. & Oomah B. D. Antioxidant activity and total phenolics in selected fruits, vegetables, and grain products. J. Agric. Food. Chem. 46, 4113–4117 (1998).

[b50] Krygier K., Sosulski F. & Hogge L. Free, esterifed, and insoluble bound phenolic acids. 1. Extraction and purification procedure. J. Agric. Food. Chem. 30, 330–334 (1982).

[b51] Christie W. W. A simple procedure for rapid transmethylation of glycerolipids and cholesteryl esters. J. Lipid Res. 23, 1072–1075 (1982). 6897259

[b52] Aslam J., Mohajir M. S., Khan S. A. & Khan A. Q. HPLC analysis of water-soluble vitamins (B1, B2, B3, B5, B6) in *in vitro* and *ex vitro* germinated chickpea (*Cicer arietinum* L.). Afr. J. Biotechnol. 7, 2310–2314 (2008).

[b53] Association of Official Analytical Chemists. *Vitamin C (ascorbic acid) in vitamin preparations and juices.* In, *AOAC Official Methods of Analysis,* (ed Elrich, K.) AOAC Official Method 967.21., 15th ed., 1058-1059 (AOAC International, Arlington, VA, 1990).

[b54] Official Journal of the European Communities. Commission Directive 2000/45/EC of 6 July 2000 establishing community methods of analysis for the determination of vitamin A, vitamin E and tryptophan in feedstuffs. L 174, 45-50. (Official Journal of the European Communities Eur-Lex, 2000).

[b55] Association of Official Analytical Chemists. *Vitamin K in milk and infant formulas.* In, *AOAC Official Methods of Analysis,* (ed Horwitz, W.) AOAC Official Method 999.15., 18th ed., 32-34 (AOAC International, Gaithersburg, MD, 2006).

[b56] Association of Official Analytical Chemists. *Metals in plants and pet foods. Atomic absorption spectrophotometric method.* In, *AOAC Official Methods of Analysis,* (ed Latimer, G.W. Jr.) AOAC Official Method 975.03., 19th ed., Ch. 3, 2–15 (AOAC International, Gaithersburg, MD, 2012).

[b57] Allain C. C., Poon L. S., Chan C. S., Richmond W. F. P. C. & Fu P. C. Enzymatic determination of total serum cholesterol. Clin. Chem. 20, 470–475 (1974). 4818200

[b58] Lopes-Virella M. F., Stone P., Ellis S. & Colwell J. A. Cholesterol determination in high density lipoproteins separated by three different methods. Clin. Chem. 23, 882–884 (1977). 192488

[b59] Schettler G. & Nussel E. Method for triglycerides. Aeb. Med. Soz. Med. Prav. Med. 10, 25–29 (1975).

[b60] Kind P. R. H. & King E. J. Estimation of plasma phosphatase by determination of hydrolysed phenol with amino-antipyrine. J. Clin. Pathol. 7, 322–326 (1954). 13286357 10.1136/jcp.7.4.322 PMC1023845

