# Supplementary material for: Nutritional properties of the largest bamboo fruit Melocanna baccifera and its ecological significance
Source: Sci Rep. 2016 May 19;6:26135. doi: 10.1038/srep26135 (PMC4872145; doi:10.1038/srep26135)
Supplement: Supplementary Information [file srep26135-s1.doc]

**SUPPLEMENTARY INFORMATION**

**Nutritional properties of the largest bamboo fruit *Melocanna baccifera* and its ecological significance**

**Balaji Govindan^1^, Anil John Johnson^1^, Sadasivan Nair Ajikumaran Nair^1^, Bhaskaran Gopakumar^2^, Mallampalli Sri Lakshmi Karuna^3^, Ramaswamy Venkataraman^4^, Konnath Chacko Koshy^2^ & Sabulal Baby^1,*^**

^1^Phytochemistry and Phytopharmacology Division, Jawaharlal Nehru Tropical Botanic Garden and Research Institute, Pacha-Palode, Thiruvananthapuram 695 562 Kerala, India, ^2^Plant Genetic Resources Division, Jawaharlal Nehru Tropical Botanic Garden and Research Institute, Pacha-Palode, Thiruvananthapuram 695 562 Kerala, India, ^3^Centre for Lipid Research, CSIR-Indian Institute of Chemical Technology, Hyderabad 500 607, India, ^4^Department of Chemistry, Sri Paramakalyani College (Manonmaniom Sundaranar University, Tirunelveli), Alwarkurichi 627 412, Tamil Nadu, India

Correspondence and requests for materials should be addressed to S.B. (sabulal@jntbgri.res.in; sabulal@gmail.com)

***Melocanna baccifera* is a unique bamboo which produces the largest fruits in the grass family. Its gregarious flowering once in 45-50 years in north east India and adjacent regions is a botanical enigma, resulting in a glut of fruits. Proper utilization of *M. baccifera* fruits is not extant, and huge quantities of fruits are left underexploited due to lack of scientific information on their chemical composition and nutritional potential. Here we report the nutritional properties of *M. baccifera* fruits, and the ecological significance of its fruiting. This pear-shaped, fleshy bamboo fruit is rich in amino acids (lysine, glutamic acid), sugars (sucrose, glucose, fructose) and phenolics (ferulic acid). Protein content (free, bound) in *M. baccifera* fruits is very low. Fruits are rich in saturated fatty acids (palmitic acid), minerals (potassium), and only B series vitamins (B3) are detected in them. Rat feeding experiments showed that *M. baccifera* fruit alone is not a complete food, but with other protein supplements, it is a valuable food additive. This study could lead to better utilization of *M. baccifera* fruits during future flowering/fruiting events. These results could also help in the successful management of rodent outbreaks and other ecological problems associated with *M. baccifera* fruiting.**

**Details of *Melocanna baccifera* (Roxb.) Kurz clumps from which fruit samples were collected**

***Melocanna baccifera*** (Roxb.) Kurz belongs to the family ***Poaceae*** (subtribe *Melocanninae*, tribe *Bambuseae*, subfamily ***Bambusoideae***)^1^.

**Live collections of *Melocanna baccifera* (Roxb.) Kurz in JNTBGRI Bambusetum**

JNTBGRI Bambusetum holds 11 *Melocanna baccifera* clumps^2^, and samples from five clumps (Accession numbers 58, 359, 365, 394, 403) were collected for the present study.

**Location in the Bambusetum, Propagule and Provenance of the five clumps studied**

***Clump Number* 58**: JNTBGRI Bambusetum, Coordinates: N 08˚45. 305’ E 077˚ 01. 491’. K. C. Koshy collected the propagule (offset) of this plant from Forest Research Institute (Coordinates: N 30.3421° E 77. 9973°), New Forest, Dehra Dun (altitude above 435 m MSL), Uttarakhand state, India on 5. 9. 1988 and planted in Institute Bambusetum on 20. 9. 1988 (Collection Number *K. C. Koshy 4330*).

***Clump Number* 359:** JNTBGRI Bambusetum, Coordinates: N 08˚45. 347’ E 077˚01. 527’. K. C. Koshy collected the propagule (offset) of this plant from Tupul (altitude 425 m above MSL), Tamenglong district, Manipur state, India on 18. 3. 1996, and planted in Institute Bambusetum on 22. 3. 1996 (Collection Number *K. C. Koshy 28606*).

***Clump Number* 365:** JNTBGRI Bambusetum, Coordinates: N 08˚45. 295’ E 077˚01.580’. K. C. Koshy collected the propagule (offset) of this plant from Saikulhills (altitude 1061 m above MSL), Senapati district, Manipur state on 28. 3. 1996 and planted in Institute Bambusetum on 30. 3. 1996 (Collection Number *K. C. Koshy 28666*)*.*

***Clump Number* 394:** JNTBGRI Bambusetum, Coordinates: N 08˚45.351’ E 077˚01.518’. K. C. Koshy collected the propagule (offset) of this plant from Lukram Lerik, Imphal district, **(**altitude 790 m above MSL), Manipur state, India on 17. 3. 1996 and planted in Institute Bambusetum on 18. 8. 1996 (Collection Number *K. C. Koshy 28607*).

***Clump Number* 403:** JNTBGRI Bambusetum, Coordinates: N 08˚45. 295’ E 077˚01. 574’. K. C. Koshy collected the propagule (offset) of this plant from Moreh (altitude 200 m above MSL), Chandel district, Manipur state on 26. 3. 1996 and planted in Institute Bambusetum on 18. 8. 1996 (Collection Number *K. C. Koshy 28648).*

**References**

1. Kellogg, E.A. *Flowering Plants. Monocots, The Families and Genera of Vascular Plants 13* (Springer International Publishing, Switzerland, 2015).
2. Koshy, K.C. *Bamboos at TBGRI* (Tropical Botanic Garden and Research Institute, Thiruvananthapuram, 2010).

**Herbarium specimens:** India, Kerala, *Thiruvananthapuram, Palode, JNTBGRI Bambusetum, accession number* **58**: 23.10.2009, *B. Gopakumar 66813*; 18.5.2011 *B. Gopakumar 66895.* **359**: 13. 7. 2007, *B. Gopakumar 47940*; 18. 11. 2008, *B. Gopakumar 47974*. **365**: 25. 11. 2009, *A. Sebastian 66645.* **366**: 25. 11. 2009, *A. Sebastian 66646*. **395**: 18. 11. 2009, *B. Gopakumar 66636.* **403**: 2. 11. 2009, *A. Sebastian 66621*. Sikkim**:** 22.2.2011, *K. C. Koshy 66689*.

**Spirit collections:** India, Kerala, *Thiruvananthapuram, Palode, JNTBGRI Bambusetum, accession number* **359:** 13. 7.2007, *B. Gopakumar 47940*; 18. 11. 2008, *B. Gopakumar 47974*; 22. 4. 2009, *B. Gopakumar 48000*; *B. Gopakumar s.n.* **394**: 27. 6. 2006, *K. C. Koshy 54179*.

---------------------------------------------------------------------------------------------------------------------


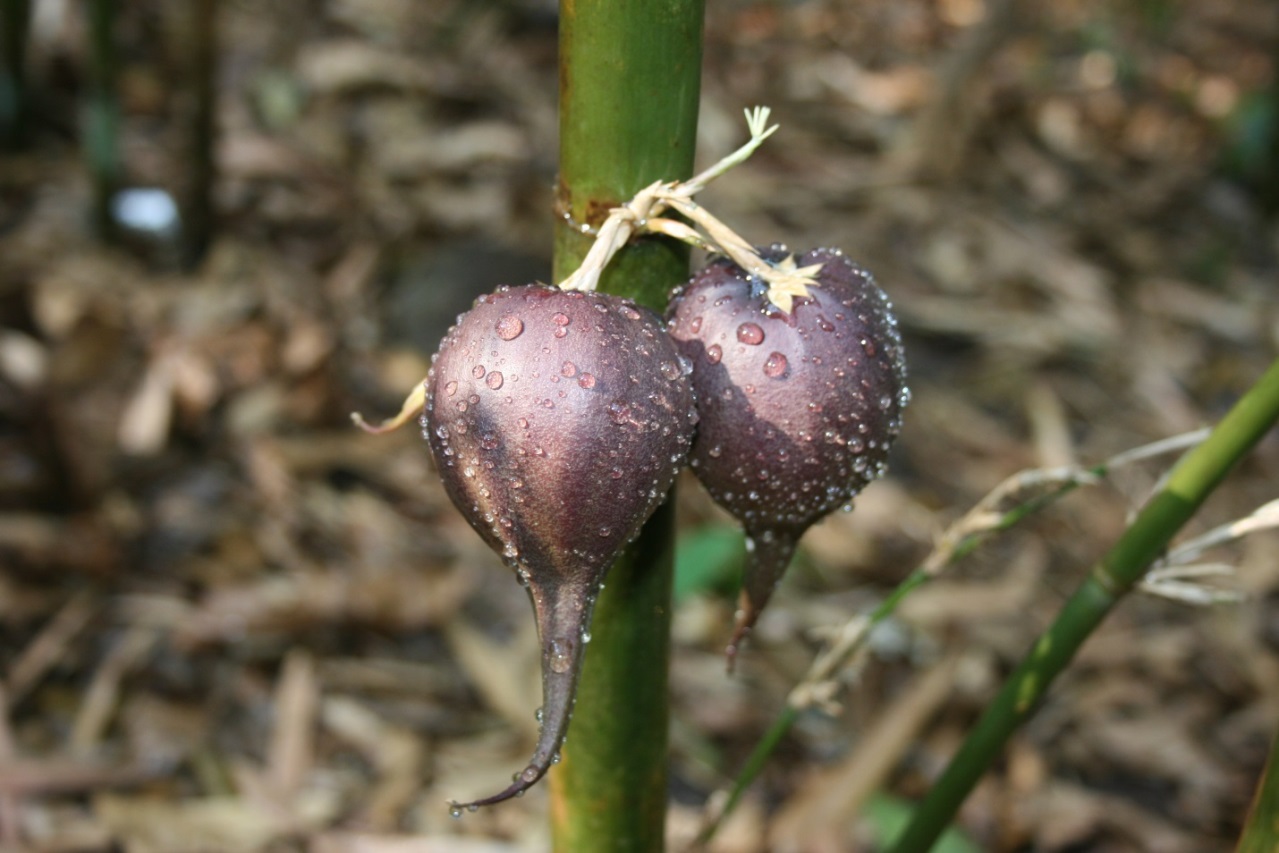


(**a**)


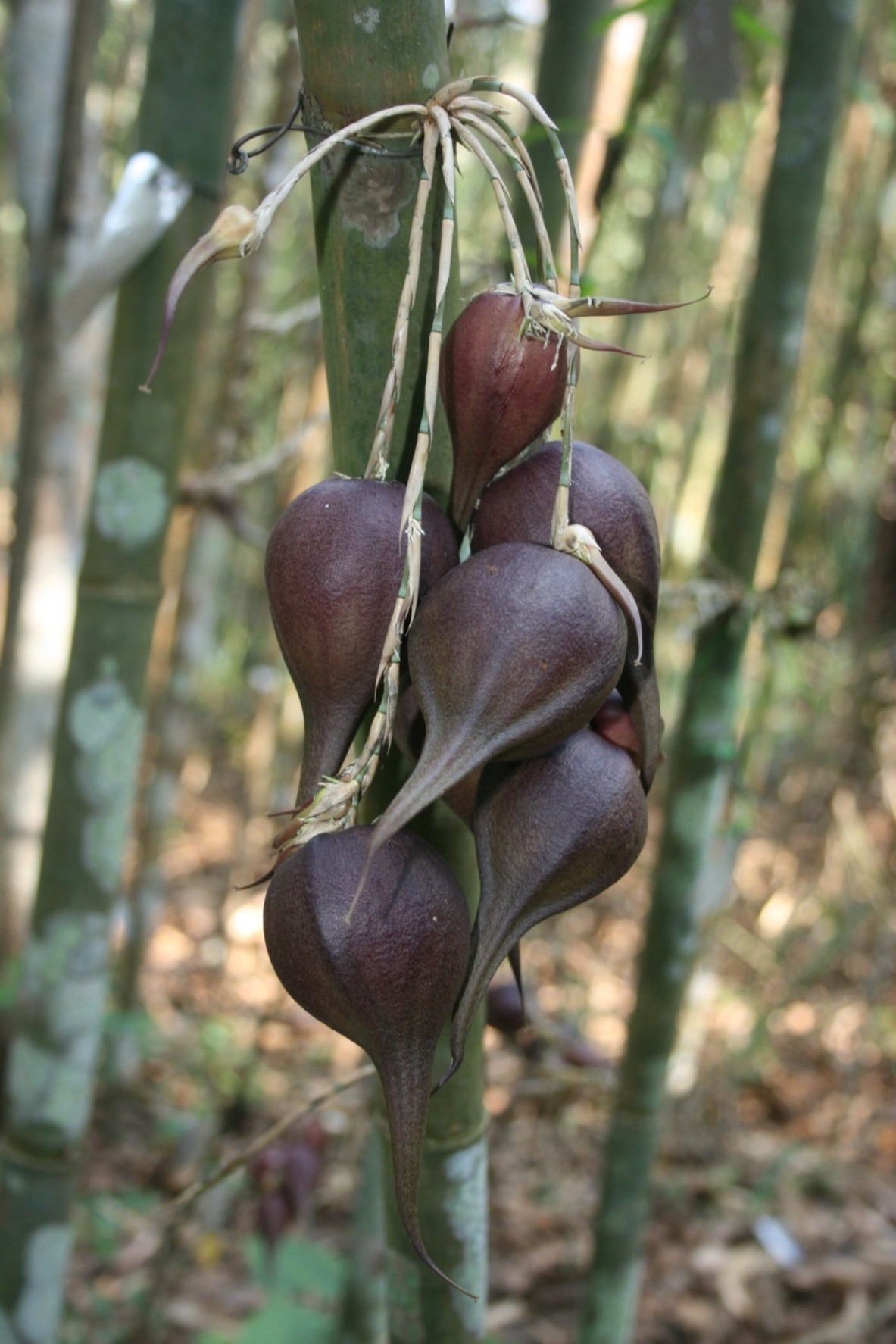


(**b**)


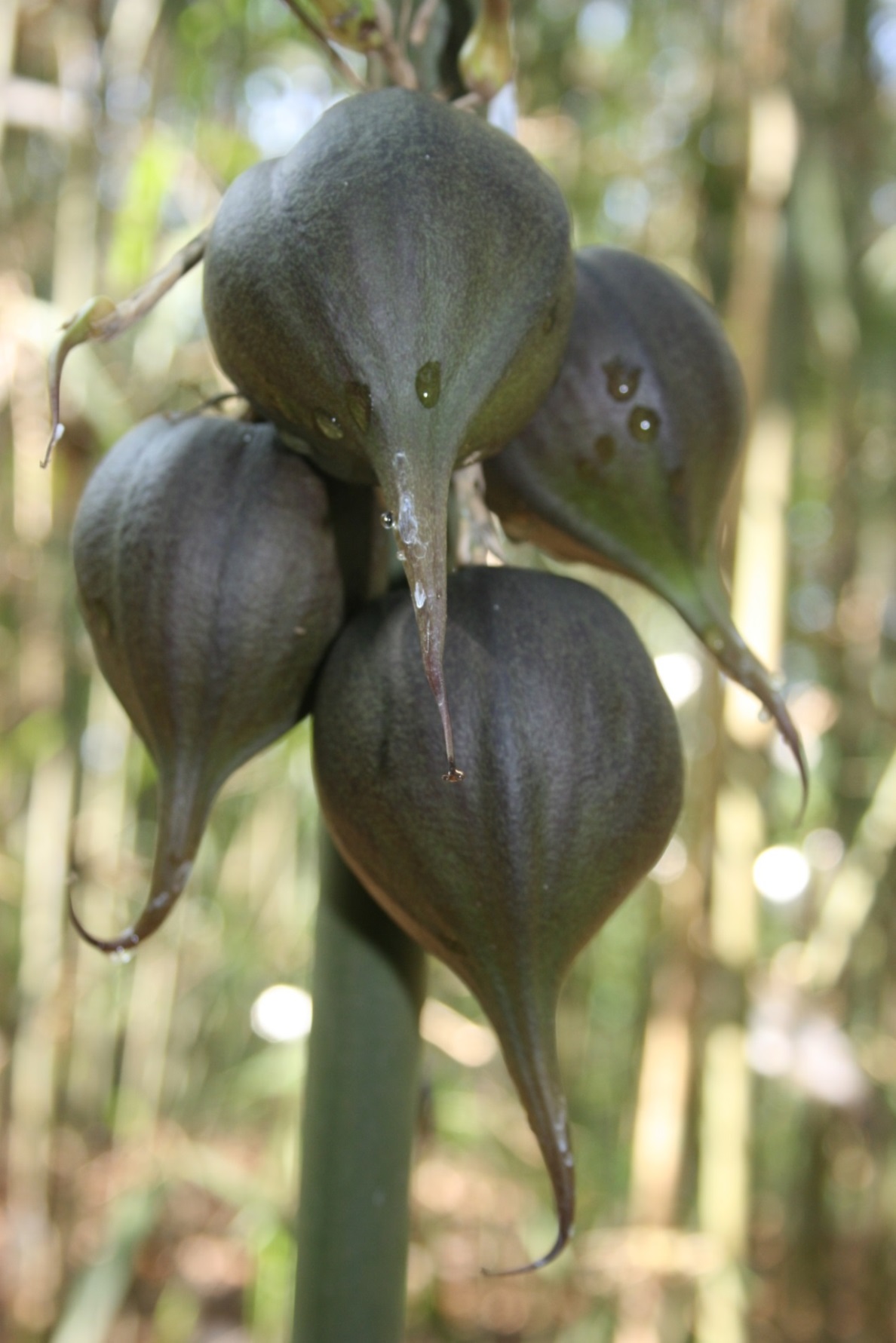


(**c**)


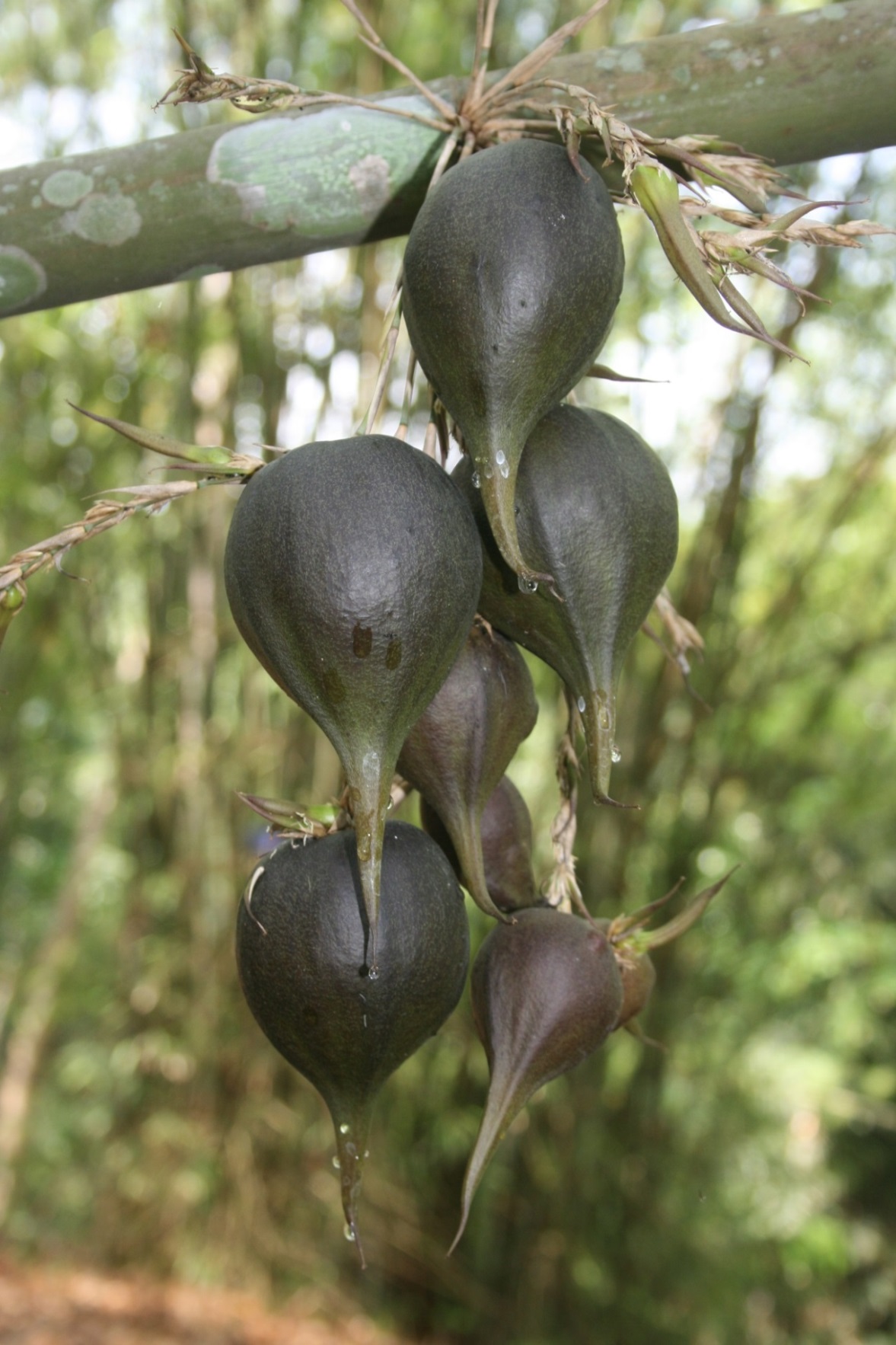


(**d**)


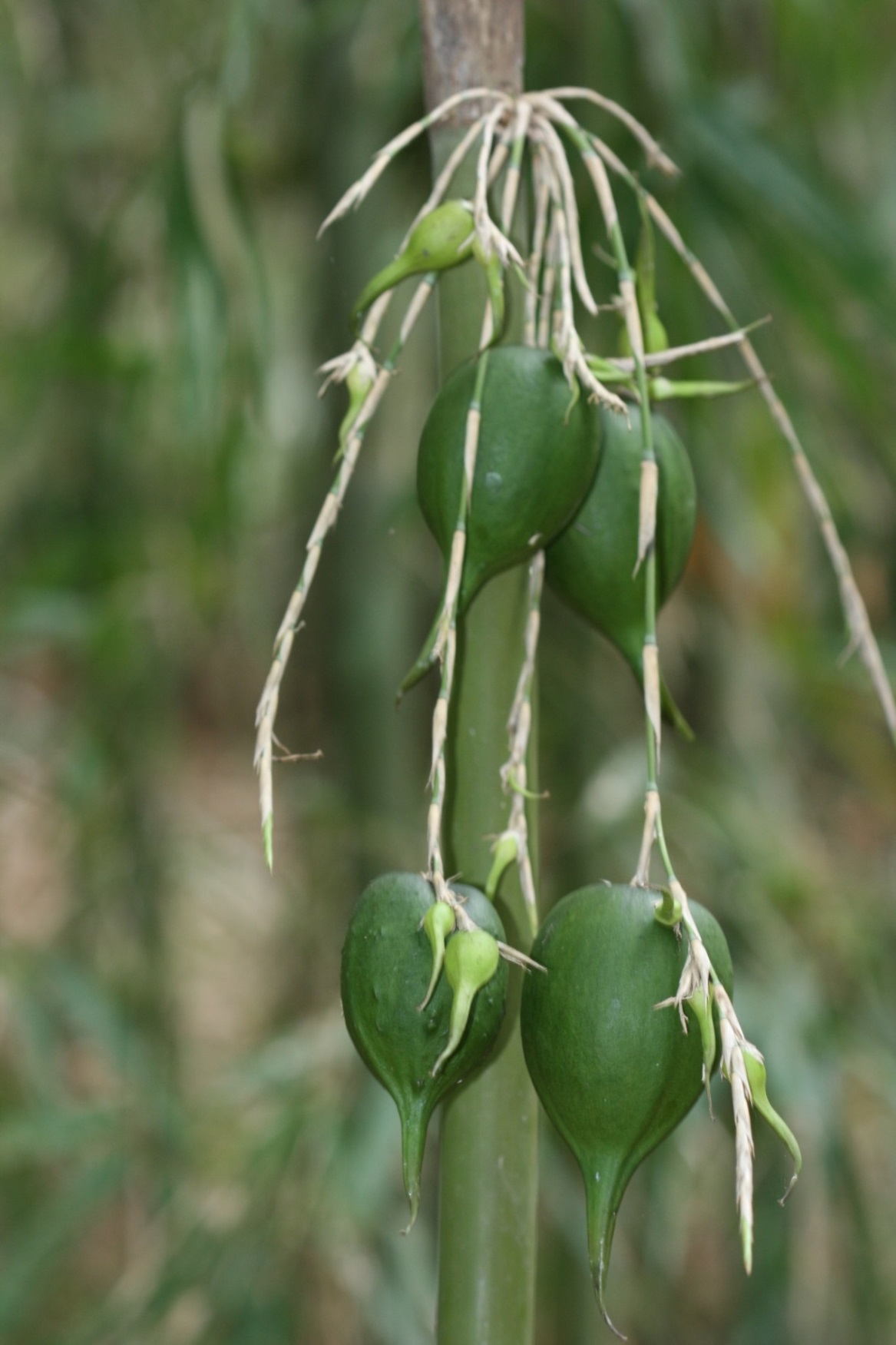


(**e**)

Fig. S1. (**a**-**e**) *Melocanna baccifera* fruits. (**a**-**c,** photographed by Bhaskaran Gopakumar; **d**-**e**, photographed by Konnath Chacko Koshy).
